# Management of uncomplicated malaria in febrile under five-year-old children by community health workers in Madagascar: reliability of malaria rapid diagnostic tests

**DOI:** 10.1186/1475-2875-11-85

**Published:** 2012-03-25

**Authors:** Arsène Ratsimbasoa, Harintsoa Ravony, Jeanne-Aimée Vonimpaisomihanta, Rogelin Raherinjafy, Martial Jahevitra, Rabenja Rapelanoro, Jean De Dieu Marie Rakotomanga, Denis Malvy, Pascal Millet, Didier Ménard

**Affiliations:** 1 Ministère de la Santé, du Planning Familial et de la Protection Sociale, Programme National de Lutte contre le Paludisme, Ministry of Public Health, BP 1869 Antananarivo, Madagascar; 2 Malaria Unit Research, Institut Pasteur de Madagascar, Antananarivo, Madagascar; 3 Université d'Antananarivo, Antananarivo, Madagascar; 4 Centre René Labusquière, Université Victor Segalen Bordeaux 2, Bordeaux, France; 5 EA Pharmaceuticals and Analytical Methods for Neglected diseases and counterfeit Drugs, Bordeaux 2 University, Bordeaux, France; 6 Malaria Molecular Epidemiology Unit, Institut Pasteur du Cambodge, 5 Boulevard Monivong, PO Box 983, Phnom Penh, Cambodia

## Abstract

**Background:**

Early diagnosis, as well as prompt and effective treatment of uncomplicated malaria, are essential components of the anti-malaria strategy in Madagascar to prevent severe malaria, reduce mortality and limit malaria transmission. The purpose of this study was to assess the performance of the malaria rapid diagnostic tests (RDTs) used by community health workers (CHWs) by comparing RDT results with two reference methods (microscopy and Polymerase Chain Reaction, PCR).

**Methods:**

Eight CHWs in two districts, each with a different level of endemic malaria transmission, were trained to use RDTs in the management of febrile children under five years of age. RDTs were performed by CHWs in all febrile children who consulted for fever. In parallel, retrospective parasitological diagnoses were made by microscopy and PCR. The results of these different diagnostic methods were analysed to evaluate the diagnostic performance of the RDTs administered by the CHWs. The stability of the RDTs stored by CHWs was also evaluated.

**Results:**

Among 190 febrile children with suspected malaria who visited CHWs between February 2009 and February 2010, 89.5% were found to be positive for malaria parasites by PCR, 51.6% were positive by microscopy and 55.8% were positive by RDT. The performance accuracy of the RDTs used by CHWs in terms of sensitivity, specificity, positive and negative predictive values was greater than 85%. Concordance between microscopy and RDT, estimated by the Kappa value was 0.83 (95% CI: 0.75-0.91). RDTs stored by CHWs for 24 months were capable of detecting *Plasmodium falciparum *in blood at a level of 200 parasites/μl.

**Conclusion:**

Introduction of easy-to-use diagnostic tools, such as RDTs, at the community level appears to be an effective strategy for improving febrile patient management and for reducing excessive use of anti-malarial drugs.

## Background

In most countries with endemic malaria, the World Health Organization (WHO) recommends using rapid and reliable diagnostic tests as an essential means of reducing the morbidity and mortality due to malaria, especially in rural areas and for children under five years of age and pregnant women [1]. The diagnosis of malaria for a long time, especially at the community level, was based solely on clinical symptoms (especially fever) [2,3], which has caused febrile patients to be misdiagnosed and inappropriately treated [4]. Thus, efficient and easy-to-use diagnostic tools should improve febrile patient management and reduce needless anti-malarial drug therapy. In addition, these tools have the potential to lower overall treatment costs and limit the selection pressure exerted by available drugs on the parasite pool. These issues continue to be relevant today despite significant decreases in malaria in endemic areas due to the implementation of effective strategies such as long-lasting bed nets (LLNs) and artemisinin-combination therapy (ACT) [5,6].

To date, microscopic examination remains the gold standard for malaria diagnosis despite its requirements for high-quality microscopes and equipment and for qualified personnel. It is clear that in endemic countries, many rural health care facilities lack the capacity to make accurate microscopic diagnoses [7,8]. Recent WHO recommendations [9,10] propose that a parasitological diagnosis be made for each individual suspected to have malaria (regardless of age group) before beginning ACT treatment. Thus, rapid diagnostic tests (RDTs) for malaria appear to be a good strategy for meeting these WHO recommendations in areas lacking either microscopy equipment or sufficiently skilled technicians.

Several immuno-chromatographic tests for detecting specific *Plasmodium *antigens are currently available. Some of these tests are specific for *Plasmodium falciparum *and detect histidine-rich protein 2 (HRP2). Others combine HRP2 detection with the detection of antigens common to all species, such as lactate dehydrogenase or aldolase (combo RDTs). Combo RDTs can diagnose infections by *P. falciparum *or by non-*P. falciparum *malaria parasites (*Plasmodium vivax, Plasmodium ovale *and *Plasmodium malariae*).

In 2005, the National Malaria Control Programme (NMCP) of Madagascar revised its national malaria treatment guidelines and replaced chloroquine with artesunate/amodiaquine combination. This change was followed in 2007 by the introduction of RDTs (CareStart Malaria™, AccessBio^®^, USA) in health care facilities lacking microscopic diagnostic capability. At the community level, the same recommendations for the treatment of fevers in children under five were put in place several years later. Finally, in 2010, in its effort to improve the management of fever at the community level, the NMCP proposed that RDTs be used by community health workers (CHWs) to diagnose febrile individuals. To do so, the NMCP set up a series of studies to validate this strategy and to answer several key questions regarding the choice of RDT, the cost of this strategy, and the establishment of a RDT quality assurance system. Although the use of RDTs requires basic training for CHWs, RDT performance (in terms of sensitivity and specificity) in the hands of CHWs and the quality of RDTs stored in the field over time have not been evaluated. In this context, the results of a longitudinal study to evaluate the overall performance and quality of RDTs used by CHWs in two regions of Madagascar with endemic malaria: one with stable malaria (the Manakara District, on the eastern coast) and the other with unstable malaria (the Moramanga District, on the edge of the central highlands) are reported here

## Methods

### Study site and participants

This study was conducted between February 2009 and February 2010 in parallel with another study (February 2008 - February 2010) that evaluated the NMCP's community level recommendations for the treatment of fevers with a fixed combination of artesunate plus amodiaquine in children under five in a region of high malaria transmission (Manakara District, the communities of Ampasimanjeva and Vohimasy) and in a region of low malaria transmission (Moramanga District, the communities of Ampasimpotsy and Andasibe) [11]. RDTs were distributed to eight CHWs (two per municipality) and the performance of the RDTs administered by the CHWs was assessed. Systematic parasitological diagnoses were performed in children who consulted CHWs for fever. This included the preparation of a thick and thin blood smears for microscopic examination/diagnosis, diagnosis by RDT, and collecting a drop of blood on filter paper for species-specific diagnostic PCR. All children under five were identified in the four communes of the two districts. All children fulfilling the following criteria were included: i) children aged two to 59 months at the time of the census; ii) children residing permanently in the community agent's study zone; and iii) children whose parents or guardians agreed to sign an informed consent at the beginning of the study.

In each village, a CHW had to manage about 150 children. CHWs in the study were volunteers who were appointed by the villagers. The CHWs represented a functional relay among the health authorities, NGOs and villagers. The level of training of the CHWs consisted of basic reading and writing capabilities, which were between the third- and fifth-grade educational levels. CHWs in the Moramanga district were supervised by the NGO "ADRA Madagascar" and by staff of the nearest health centre. They had previously received regular training on how to care for fevers at the community level. In exchange for their participation, CHWs received food staples (oil, rice). CHWs in Vohimasy and Manakara, were supervised by the NGO "Inter Aide" under the name of "Maman et Papa conseils." These CHWs had also received training on how to care for fevers at the community level. CHWs in Ampasimanjeva were supervised by the NGO "RTM". In these two communities, agents received help from the villagers to maintain their fields and an annual allocation of rice as compensation for their work. At the beginning of the study, each CHW received a three-day training session on the general and clinical aspects of malaria, on the use of thermometer, the preparation of thick and thin blood smears, and on collecting blood spots on filter paper. Special training was provided for use of the CareStart RDT and the interpretation of the test results.

### Blood sampling and diagnosis

All children in the study were two months of age or more and had experienced a fever with the following criteria: axillary temperature of 37.5°C or fever for at least 24 hours. Children with signs of severe malaria or other infections that were life threatening were excluded from the study and referred to the nearest health centre. At each visit, CHWs collected a finger-prick blood sample to prepare a thin/thick smear and a blood spot. They also performed an RDT according to the instructions received. The samples were then stored at room temperature and protected from light. The results of the RDTs were recorded in a book provided for that purpose. Regardless of RDT outcome, all children were treated with a fixed dose of artesunate/amodiaquine. Every months (each supervisory visit), slides, RDTs and blood spots were transferred to the Malaria Research Unit of Institut Pasteur de Madagascar (IPM).

### Microscopic examination of slides

Suitable slides were stained and read by two experienced IPM microscopists. Briefly, after the smear was fixed in methanol, the slide was stained with 10% Giemsa for 10 min, dried, and then examined by microscopy at 100× with an immersion objective. Parasite density (i.e., the number of parasites per μL) was on the number of asexual parasites per 200 white blood cells (WBC) counted (or per 500 WBC if the parasite count was less than 10 parasites per 200 white blood cells). Parasitaemia was expressed in terms of the number of parasites per μL, considering that the average concentration of WBCs is 8,000 per μL. The smear was declared negative if no parasites were observed after reading 100 microscopic fields. The thin film was used to identify the species of the parasite(s). The slides were read independently by two microscopists. The final parasite density was calculated from the mean estimated parasite densities. For cases in which the difference between the two density estimates was greater than 10%, a third reading was performed by a third microscopist. Quality control was also provided by technicians at the NMCP, who analysed 10% of the slides.

### DNA extraction and PCR diagnosis of malaria

DNA was extracted from blood spots using Instagene matrix (BioRad, Marnes la Coquette, France) according to the manufacturer's instructions. The detection of malaria parasites was assessed by real-time PCR using methods previously described by using RotorGene 3000 thermocycler (Corbett Life Science, Sydney, Australia) [12]. PCR analyses were performed by technicians blinded to the results of microscopic diagnoses.

### Quality control of rapid diagnostic tests

The long-term stability of RDTs kept by CHWs was evaluated according to the protocol developed by WHO/FIND/TDR described in the "Methods manual for laboratory quality control testing of malaria rapid diagnostic tests" [13]. Briefly, initial RDT quality control was conducted in February 2008 (before the RDTs were implemented) and every six months thereafter until the end of the study, on RDTs collected from CHWs involved in the project. RDT quality was assessed using calibrated aliquots of *P. falciparum *and *P. vivax *(at 2,000 and 200 parasites per μL) and negative controls.

### Data analysis

All data were recorded and analysed using EpiInfo software (version 3.3.2, CDC, Atlanta, GA, USA) and MedCalc (MedCalc Software, Broekstraat 52, 9030 Mariakerke, Belgium). A chi-squared test was used to compare the performances of the different diagnostic methods used (PCR, microscopy and RDTs). A P-value < 0.05 was considered to indicate statistical significance. To evaluate the sensitivity and specificity, the microscopy and RDT results were compared with those of PCR. Sensitivity was calculated as the proportion of positive tests recorded for the samples found positive by the reference method chosen. Specificity was estimated as the proportion of negative tests recorded for samples found negative by the reference method chosen. The positive and negative predictive values (PPV and NPV, respectively) were also calculated as the proportion of results that were "truly positive" or "truly negative" among the samples found to be positive or negative using the reference method chosen. Finally, Kappa values were calculated to estimate the correlation between diagnostic techniques. Kappa values between 0.21 and 0.60 were considered to indicate "moderate agreement"; values between 0.61 and 0.80 indicated a "good fit"; and values greater than 0.80 indicated "excellent agreement".

### Ethics component

The study was conducted in compliance with the Helsinki Declaration and the laws of Malagasy. The protocol was submitted and approved by the Ethics Committee of National Malagasy (No. 153/SANPF/2007). Signed informed consent was obtained from the parents or legal guardian of the child. If parents or guardians could not write, a fingerprint was made. If parents or caregivers could not read, consent information was read to them in their local dialect. In this case, a witness was required to be present to sign the consent. This protocol was recorded in the registry of clinical trials at http://www.clinicaltrials.gov and its identification number is NCT00612547.

## Results

### Patient enrolment

Between February 2008 and February 2010, 1,073 children were enrolled in the cohort study as described elsewhere [11]. Of these, 543 febrile episodes were referred to CHWs: 422 children presented a single episode during the study period, 99 presented two episodes, 19 had three episodes, two had four episodes and one had five episodes. No significant differences in the relative number of episodes were noted between the four areas (Ampasimanjeva, n = 309; Vohimasy, n = 118; Ampasimpotsy, n = 63; and Andasibe, n = 53; P = 0.8). Similarly, no significant sex ratio differences were found between the municipalities. In contrast, the average age of the febrile children was significantly higher in areas of low transmission than in areas of high transmission (Table 1).

**Table 1:** Characteristics of patients included in the study, Madagascar, 2008-2010

Characteristics	Study sites				*P*
		
	High transmission		Low transmission		
		
	Ampasimanjeva	Vohimasy	Ampasimpotsy	Andasibe	
Cohort population	281	258	299	235	

Mean age (months)	28.6	26.5	30.1	31.5	0.009

Sex ratio (M/F)	1	0.87	0.86	0.94	NS

No. of febrile episodes	309	118	63	53	

PCR + (%)	56%	58%	33%	30%	< 0.0001

Microscopy + (%)	37%	43%	32%	25%	NS

## PCR results

Of the 529 blood spots collected from February 2008 to February 2010 (97.4% of febrile episodes), 271 (51.2%) were positive only for *P. falciparum*. The percentage of children with fever and parasite carriers was approximately two times higher in areas of high transmission (56.7% vs. 31.9%, P < 0.0001) (Tables 1, 2, 3 and 4).

**Table 2:** Comparison of malaria test results according to the diagnostic methods used for all sites combined in Madagascar, 2008-2010

Nested PCR	n (%)	Microscopy		RDT			Microscopy	RDT			
		
Results		Negative	*Pf*	Negative	HRP2+	HRP2/pLDH+	Results	n (%)	Negative	HRP2+	HRP2/pLDH+
Negative	258 (48.8)	258	0	19	1	0	Negative	92 (48.4)	80	7	5

*Pf*	271 (51.2)	74	197	65	11	94	*Pf*	98 (51.6)	4	5	89

**Table 3:** Comparison of malaria test results according to the diagnostic methods used for the high-transmission areas in Madagascar, 2008-2010

Nested PCR	n (%)	Microscopy		RDT			Microscopy	RDT			
		
Results		Negative	*Pf*	Negative	HRP2+	HRP2/pLDH+	Results	n (%)	Negative	HRP2+	HRP2/pLDH+
Negative	179 (43.3)	179	0	18	0	0	Negative	86 (51.2)	75	6	5

*Pf*	234 (56.7)	70	164	60	11	79	*Pf*	82 (48.8)	3	5	74

**Table 4:** Comparison of malaria test results according to the diagnostic methods used for the low-transmission areas in Madagascar, 2008-2010

Nested PCR	n (%)	Microscopy		RDT			Microscopy	RDT			
		
Results		Negative	*Pf*	Negative	HRP2+	HRP2/pLDH+	Results	n (%)	Negative	HRP2+	HRP2/pLDH+
Negative	79 (68.1)	79	0	1	1	0	Negative	6 (27.3)	5	1	0

*Pf*	37 (31.9)	4	33	5	0	15	*Pf*	16 (72.7)	1	0	15

### Microscopy results

Among the 543 slides collected, only 14 slides (2.6%) were not suitable for microscopy examination. One hundred ninety seven (37.2%) were positive for *P. falciparum*, which was the only species found. The percentage positive slides did not differ by location. Parasite densities for the positive slides were between 48 and 82,000 parasites per μL. There were no significant differences between the mean parasite densities found in each area (Tables 1, 2, 3 and 4).

### Rapid diagnostic test results

Of the 190 RDTs carried out by CHWs between February 2009 and February 2010, 106 (55.8%) were positive: 94 tests detected both antigens (HRP2 and pLDH) and 12 tests detected only the HRP2 antigen (Tables 2, 3, and 4). Compared to the PCR results, the RDTs produced 124 (65.3%) matching results and 66 (34.7%) discordant results (98.5% were false negatives and 1.5% were false positives). The RDT results for the two transmission zones were similar. In the high transmission zone, 64.3% of the RDT results matched those of the PCR analysis whereas 35.7% did not (100% were false negatives). In the low transmission zone, 72.7% of the RDT results agreed with those of PCR and 27.3% rendered either false negatives (83.0%) or false positives (17.0%). All false-positive RDTs consisted of the HRP2 band only.

Compared to the microscopy analysis, the RDTs produced 174 (91.6%) concordant results and 14 (8.4%) discordant results (25% were false negatives and 75% were false positives). The RDT results were similar for the two transmission zones. For the high transmission zone, 91.6% of the RDT results agreed with the microscopy results 8.4% did not (21.4% were false negatives and 78.6% were false positives). For the low transmission zone, 88.8% of the RDT results were agreed with the microscopy results and 11.2% were discordant (50.0% were false negatives and 50.0% were false positives). Only six of 11 false-positives RDTs produced only the HRP2 band. Four false-negative RDTs corresponded to samples in which the parasite levels were below 1,000 parasites/μL.

### Performance of rapid diagnostic tests performed by community health workers

RDT performance was evaluated by using either the PCR or microscopy results as reference standards. The analysis was done according to the two transmission zones (Tables 5 and 6). Overall, the sensitivity and the NPV of the RDTs were low compared to the PCR method. However, the specificity and PPV were close to 100%. This trend was found for both transmission areas, although the number of RDT results for the low transmission area was too low to determine specific outcomes. Compared to microscopy, the performance values (sensitivity, specificity, Positive Predictive and Negative Predictive Values) for the CHW-administered RDTs were all above 85%. Finally the Kappa values obtained for comparisons between the three different methods reflected a good correlation between PCR and microscopy (0.72; 95% CI: 0.66 to 0.78) and between microscopy and the RDT (0.83, 95% CI: 0.75-0.91). In contrast, the Kappa value was very low between PCR and RDT (0.23, 95% CI: 0.14-0.33).

**Table 5:** Sensitivity, specificity, positive predictive values (PPV) and negative predictive values (NPV) of microscopy and RDTs *versus *PCR (reference method) in high- and low-transmission areas in Madagascar, 2008-2010

Global	Methods	
	
	Microscopy	RDT
Sensitivity (95% CI)	72.7 (67.0-77.9)	61.8 (54.0-69.1

Specificity (95% CI)	100.0 (98.5-100.0	95.0 (75.1-99.9)

PPV (95% CI)	100.0 (98.1-100.0)	99.0 (94.9-100.0)

NPV (95% CI)	77.7 (72.8-82.1)	99.0 (94.9-100.0)

High transmission	Methods	
	
	Microscopy	RDT

Sensitivity (95% CI)	70.1 (63.8-75.9)	60.0 (51.7-67.9)

Specificity (95% CI)	100.0 (97.9-100.0)	100.0 (81.5-100.0

PPV (95% CI)	100.0 (97.8-100.0)	100.0 (96.0-100)

NPV (95% CI)	71.9 (65.9-77.4)	23.1 (14.3-34.0

-Low transmission	Methods	
	
	Microscopy	RDT

Sensitivity (95% CI)	89.2 (74.6-97.0)	75.0 (50.9-91.3)

Specificity (95% CI)	100.0 (95.4-100.0	50.0 (1.3-98.7)

PPV (95% CI)	100.0 (89.4-100.0)	93.7 (69.8-99.8)

NPV (95% CI)	100.0 (89.4-100.0)	16.7 (0.4-64.1)

**Table 6:** Sensitivity, specificity, positive predictive values (PPV) and negative predictive values (NPV) of RDTs compared to microscopy (reference method) in the high- and low-transmission areas in Madagascar, 2008-2010

	Global	High transmission	Low transmission
Sensitivity (95% CI)	95.9 (89.9-98.9)	90.2 (81.7-95.7)	93.7 (69.8-99.4)

Specificity (95% CI)	87.0 (78.3-93.1)	87.2 (78.3-93.4)	83.3 (35.9-99.6)

PPV (95% CI)	88.7 (81.1-94.0)	87.1 (78.0-93.4)	72.7 (49.8-89.3)

NPV (95% CI)	95.2 (88.2-98.7)	90.4 (81.9-95.8)	93.7 (69.8-99.8)

### Stability of rapid diagnostic tests stored by community health workers

All RDT control samples were found to be compliant before the tests were distributed. After distribution, six-month quality evaluations revealed that all RDT controls were compliant prior to those tested at 18 months and 24 months. For these RDT controls, the *P. falciparum *and negative controls were compliant but the concentration for the 200 parasites/μL. *P. vivax *control was non-compliant (200 and 2000 parasites/μL).

## Discussion

In Madagascar, the integrated management of febrile syndromes at the community level remains a priority NMCP strategy for providing impoverished populations with diagnosis results and effective anti-malarial treatment within 24 hours after the febrile individual has access to care. In fact, this strategy relies on a system of CHWs and staff working in health centres, which has already proven to be effective for many years. The operational research project, evaluating the use of ACT and the implementation of RDTs, works through a network of CHWs and existing operations, which enabled us to obtain reliable data on the efficacy of "diagnostic" and "therapeutic" agents. Despite encountering difficulties in establishing a consensus for the type of RDT to be used in Madagascar, the NMCP introduced "combo" RDTs, which detect the HRP2 antigen specific for *P. falciparum *and the pLDH of four *Plasmodium *species: *P. falciparum, P. vivax, P. malariae and P. ovale *[12,14,15].

To evaluate the performance of RDTs used by CHWs, their results were compared to those obtained from the two most common methods used to diagnose malaria: PCR, which has the best performance in terms of sensitivity and specificity due to its very low threshold of detection (< 1 parasites/μL); and microscopy, which remains the WHO reference diagnostic despite its higher detection limit (10-50 parasites/μL) [16]. For the 24-month period of the study, the number of febrile episodes in children that consulted with CHWs in the two study areas of high and low transmission was not significantly different. The only notable difference was that the mean age of the febrile children seen by CHWs in the areas of low transmission was higher than that of the children in the high transmission areas (~3 months, *P *= 0.009).

From an epidemiological standpoint, malaria burdens in the two study areas were assessed through the use of PCR. On average, 51.2% of the children consulting CHWs were carriers of parasites, and this proportion in area of high transmission was nearly twice as high as that in the low-transmission area (56.7% vs. 31.9%, respectively; *P *< 0.0001). However, this difference was not evident if microscopy, which detected an infection prevalence of 37.2%, was used as the diagnostic method. This finding demonstrates the importance of using molecular methods to study malaria epidemiology [17,18]. In addition, all children with parasites were infected only with *P. falciparum*, the predominant species in Madagascar [19,20]. However, microscopy allowed measuring the extent of parasite densities (48 to 82,000 parasites/μL) in the infected children. Although the difference was not statistically significant, the mean parasite densities were higher in high-transmission areas than in low-transmission areas (2,800 parasites/μL vs 1,500-2,200 parasites/μL). This is consistent with the idea that the "pyrogenic" thresholds of individuals in high-transmission areas are higher than those in low-transmission areas, and that below this threshold, patients do not suffer from malaria "disease" [21,22].

From a practical standpoint, no major difficulties in the use of RDTs were noticed by CHWs, despite reports to the contrary [23,24]. Training before the start of the study, the availability of simple data collection tools, and the supervision of CHW activities by physician supervisors facilitated RDT implementation [25]. The simplicity of the RDT procedure has also avoided many errors of manipulation or interpretation. The few difficulties encountered by the CHWs were mainly related to the amount of blood drawn at the finger (especially for very young children) or the reading and interpretation of the results of certain tests, as has been previously noted by Seidahmed *et al *[26].

The performance of CHW-administered RDTs was determined by comparing the RDT results to those obtained by PCR and by microscopy. The levels of confirmation were similar for the CHWs in all the study areas. Discrepancies between RDT results and those of microscopy or PCR were mainly related to their different thresholds of detection as has often been observed either at the health centres [12,15,16,27,28] or at the community level [29,30]. False positives (HRP2 antigen detection only) generally corresponded to the detection of old malaria infections (and correctly treated) and were related to the persistence of the HRP2 antigen in the bloodstream (up to three weeks after malaria infection) [31,32]. Overall, the performance of RDTs used by CHWs was similar to that of microscopic analysis performed by an experienced microscopist. Compared to microscopy, RDT sensitivity was excellent (95.9%) both in high-transmission areas (90.2%) and in low-transmission areas (93.7%). However, RDT specificity relative to that of microscopy was lower (87.0%) as previously reported [33-35]. Finally, the RDTs produced an excellent NPV of 95.2%. These encouraging results justify using RDTs to diagnose malaria in areas that are most in need of low-cost diagnostic techniques.

If CHWs had only treated children found RDT-positive, only four patients who were RDT-negative and microscopy-positive, or 2% of the patients seen, did not receive anti-malarial treatment. These four patients who had very low levels of parasitaemia (less than 1,000 parasites/μL) would have been tested again a day or two later by the community worker if clinical signs had persisted. Another encouraging sign for the introduction of RDTs at the community level was the good long-term stability of the RDTs over time, given they were kept in the field conditions by the CHWs. The potential benefits of RDT-negative results (that excluded a malarial infection) could not be acted upon in the study. However, based on the PCR results, 49% of the children with fever who consulted CHWs were not infected with malaria. There were significantly more of these cases in the low-transmission areas (68.1%) than in the high-transmission area (43.3%). These data show that this testing strategy is solely limited to detect malaria infections and malaria is only a fraction of the fever etiologies seen at the community level. It is clear that further studies should be conducted to determine the agents responsible for non-malarial fever and to develop simple algorithms (clinical, biological and therapeutic, if possible) to better manage cases of non-malarial fevers.

## Conclusions

The results, report here, indicate that the management of cases of fever among children under five by CHWs is desirable and feasible in rural areas, such as Madagascar, where many people live a considerable distance from health care facilities. Although the role of CHWs in the care of patients remains a subject of debate, our study suggests RDT use by CHWs improves access to quality care for individuals with uncomplicated malaria. Such a strategy is likely to substantially reduce the prevalence of severe malaria in young children, thereby preventing many deaths, and to reduce the overuse of anti-malarial drugs, which could reduce the risk of emergence and spread of artemisinin-resistant parasites. It remains for the NMCP of Madagascar to accurately assess the costs and benefits of this strategy according to the different epidemiological patterns that exist in Madagascar, especially those of the southern desert and those of the tropical west coast. It will also be necessary to develop new studies to improve the management of non-malarial fevers that produce a negative RDT result.

## Competing interests

The authors declare that they have no competing interests.

## Authors' contributions

DM and AR contributed to the design and coordination of the study, assisted with data entry and interpretation and prepared the manuscript. HR, JAV supervised the field study. RRah and MJ were involved in laboratory work. RRap, JDMR, DMa and PM helped to compose the manuscript and gave constructive. All authors read and approved the final manuscript.
